# Supplementary material for: Patterns of Intron Gain and Loss in Fungi
Source: PLoS Biol. 2004 Nov 30;2(12):e422. doi: 10.1371/journal.pbio.0020422 (PMC532390; doi:10.1371/journal.pbio.0020422)
Supplement: Table S1 — Also available at http://genes.mit.edu/NielsenEtAl/. (4.3 MB ZIP). [file pbio.0020422.st001.zip › NielsenEtAl/html/1404.html]

AN3432.1.NCU09705.1.MG03853.1.FG06059.1


```
 CLUSTAL W (1.82) Multiple Sequence Alignments - Introns Inserted


Sequence 1: NCU09705.1	350 aa
Sequence 2: MG03853.1	342 aa
Sequence 3: FG06059.1	340 aa
Sequence 4: AN3432.1	333 aa
Alignment Length: 353 aa
Number Identitical Residues: 114 aa
Alignment Score (without introns) 6919


MG03853.1 	MSLHEPIKLLPLGAIIQSFKVGDVDIVQGFPTQELYEKHNSPYFGETIGRVANRTKDAKI
NCU09705.1	MS-DAIASFIPLGAIIQSLYVAGTNIVLGFPSQKAYESHNACFFGETIGRVANRVKGAKI
FG06059.1 	MA-DSSISFLPLGAIIQSIVVNGVNIVQGFPTQEDYEKYNYPYFGVTVGRIANRIKDARI
AN3432.1  	MGDHTSVKFSNLAAIIQEFNVDGQNIVQGFTKKEYYEKYNKPWFGATIGRVANRIKNGVI
          	*. .   .:  *.****.: * . :** **..:: **.:*  :** *:**:*** *.. *

MG03853.1 	NSLNGGKSYTLTANDGPNNLHGGVVGWGKRVWEGPKAVGIRDIPGIEG----LVGGESVE
NCU09705.1	DSLNGGQSYTLAANNGPNNLHGGVVGWGKKVWDGPKPVGVREIPAFEGKSDKIDGGESVQ
FG06059.1 	DSLNG-KEVILAANDGKNHLHGGKVGWSTRVWDGPKPVGIREVPGVEG----LEGGESVA
AN3432.1  	NNLNG-REYKLEINNPPNALHGGSRGFGRQEFEGPTVIKR-------------DGKDALL
          	:.*** :.  *  *:  * ****  *:. : ::**. :                * ::: 

MG03853.1 	FSLTSEDGDEGFPGTVKAKTIYTAG-EQTVDGKKVTVLGIEYIAELVG-DAEETPINMTN
NCU09705.1	FTLVSEDGDEGFPGKVEARVIYTVSKEKGEDGKEKVLLGIEYEAELLDGEVEETVINMTN
FG06059.1 	FTLTSEDGDEGYPGTVEVTVTYTTG-TQKVDGKDVTILAMEYEAKLVG-GADETVINMTN
AN3432.1  	YTYLSPDGDNGYPGTVELRVYYVVS-EEVEGGLPRSILNIEYEAELVGDEVEETVINITN
          	::  * ***:*:**.*:  . *...  :  .*    :* :** *:*:.. .:** **:**

MG03853.1 	HS2YFNLTGGPSIAG-TVVEIATNAYLPVDDGGIPTGGVGAFSK---FEGNKPFTLGPQE
NCU09705.1	HS2YFNLTGGPSIEG-TIITLATNQYLPVDQGGIPTGGPVPYPNPAVKNGNIPFTLGAAE
FG06059.1 	HS~YFNPSGKETIAD-TEMTLPTASYLAVGDDLIPTGKIESFPG---VTANKTFTLGPQE
AN3432.1  	HS2YFNLSGAASAAEGVTAKLTTRDYLPLENG-IPLGQISPHSI----DTTQPFEFGPDK
          	** *** :*  :   ..   :.*  **.: :. ** *   ...       . .* :*. :

MG03853.1 	PCIDDCFIANDKPESVPIDTSSLPLSRHVAAHHPDSGIHLEVYSTEPAFQFYTGKYIDVP
NCU09705.1	PDIDDCFIVNEDPSSVPIDTRSLPLKKNLSAFHPESKIHLEVYSTEPAFQFYTGKYIDIP
FG06059.1 	PNIDHCFVLNEDPSSVPLDTRSQPLNLNVKAHHAGSKINLEVYSTEPTFQIYTGAGINVP
AN3432.1  	ATFDDCFVVDRDLSGVSLDTRNRPLKLLAEFRHADTRMNLQVHSTDPAFQFYTGAGIDVQ
          	. :*.**: : . ..*.:** . **.      *. : ::*:*:**:*:**:***  *:: 

MG03853.1 	EVGG-VPARKARSAFCVEPSRYVNAVNVDEWKNMTVLKKGQKYGARIVYRAWKE
NCU09705.1	AVDG-VPARKPRSGFCLEPSRWVNACNVPEWKGQMLLKKGEKYGCKILYRAWKE
FG06059.1 	AVNG-LPARKARAGFCCEPARYVNAANVPEWKNQVLLKKGETYGARTVYKVWSD
AN3432.1  	KADEENPARGPWAGFCIEPSRYVNAINEEKWRHTVVLKKGEKYGSRIMYKAWRG
          	 .. . *** . :.** **:*:*** *  :*:   :****:.**.: :*:.*
```
